# Cognitive rehabilitation for functional neurological disorder

**DOI:** 10.1017/S1092852925100825

**Published:** 2025-12-07

**Authors:** Ryan Van Patten, Eva Keatley

**Affiliations:** 1 Center for Neurorestoration & Neurotechnology, VA Providence Healthcare System, Providence, RI, USA; 2 Department of Psychiatry and Human Behavior, Warren Alpert Medical School, Brown University, Providence, RI, USA; 3 Physical Medicine and Rehabilitation, Johns Hopkins School of Medicine, Baltimore, MD, USA

**Keywords:** Functional neurological disorder, functional cognitive disorder, cognitive rehabilitation, treatment, neuropsychology, neuropsychiatry

## Abstract

Cognitive problems represent one of the most common symptom dimensions in functional neurological disorder (FND; >80% of patients) and are frequently associated with distress, disability, and difficulties engaging in evidence-based treatments such as psychotherapy. Cognitive difficulties occur across the FND subtypes (eg, seizures, movement disorders, dizziness) but are largely underrecognized and undertreated by healthcare providers. That is, although a variety of interventions are available for primary functional symptoms and mental health comorbidities, there have not been any systematic efforts to date to specifically target cognitive functioning in FND, leaving an important gap in the literature.

Cognitive rehabilitation is a flexible approach utilizing diverse techniques aimed at improving cognition and enhancing functional independence in people with neuropsychiatric disorders. Cognitive rehabilitation can have positive impacts (moderate effect sizes) on cognition and everyday functioning across a variety of conditions, including traumatic brain injury, mild cognitive impairment, long COVID, PTSD, and others. Given the transdiagnostic clinical utility of cognitive rehabilitation, it has potential for benefit in many patients with FND if adapted and applied appropriately.

In this review, we highlight the utility of cognitive rehabilitation for FND, with a focus on clinically actionable advice and guidance. We describe fundamental principles of cognitive rehabilitation, evidence for its efficacy and effectiveness across neuropsychiatric disorders, and methods for avoiding potential pitfalls when applying it in FND. We then discuss a Case Vignette in order to emphasize the application of cognitive rehabilitation principles in an individual patient. We conclude with future directions for research and clinical care.

## Introduction

Functional neurological disorder (FND) is a common neuropsychiatric condition characterized by involuntary sensorimotor and/or cognitive difficulties that are inconsistent with known neurological diseases and are associated with neural network dysfunction rather than identifiable structural neuropathology.^1^ FND is among the most frequent disorders seen in neurology clinics worldwide, with an estimated minimum point prevalence of 80–140 per 100,000 individuals (exceeding multiple sclerosis, motor neuron disease, and brain tumors, combined).^2^ Healthcare costs are high, being estimated at €33,697 on average per year for each Danish patient with functional/dissociative seizures^3^ (FDS; €15,121 for their partners), while overall yearly costs for all patients with FND in the United States exceed $2 billion.^4^ Initial onset of functional symptoms is typically in young or middle-aged adults, although FND is a lifespan disorder, occurring in children through older adults. Symptoms are often chronic (persisting for years or decades) and difficult to treat, leading to high rates of prolonged unemployment and disability.^5^

FND can be reliably diagnosed by experienced clinicians using positive signs, reflecting a departure from an older conceptualization that treated it as a diagnosis of exclusion.^6^ By convention, FND is typically recognized and grouped by subtype, based on the primary functional presentation such as seizures, movement disorders, dizziness, or cognitive disorders, among others.^1^ Beyond the functional symptom(s), FND is associated with a wide range of risk factors and comorbidities, including other neurological disorders (eg, epilepsy, traumatic brain injury [TBI], Parkinson’s disease), psychological and mental health factors (eg, depression, anxiety, trauma, PTSD, dissociation, somatization), and systemic physical conditions (eg, insomnia, obesity, irritable bowel syndrome), among others.^7^^,^^8^ From a psychosocial standpoint, many patients with FND report experiencing stigma and discrimination from healthcare providers and the general public alike, due in part to the false belief that FND is equivalent to feigning, and/or that the symptoms are “all in the head” of the patient.^9^ Overall, contemporary approaches to assessment and treatment of FND are based primarily in the biopsychosocial model in order to account for heterogeneity and complexity in the predisposing, precipitating, perpetuating, and protective factors that affect patients’ quality of life (QoL) and treatment outcomes.^10^

## Cognition in FND

Cognitive difficulties represent one of the most common and disabling symptom dimensions across FND phenotypes and can be measured using self-report (ie, subjective or perceived concerns), performance-based testing (ie, objective deficits), or both. Regarding the former, an international survey of 1,048 people who reported having been diagnosed with FND found that memory problems were the second most common symptom (>80% of the sample), after fatigue (93%).^11^ Similarly, another study of 47 patients with video-EEG confirmed FDS and a history of TBI identified 79% of the sample (37/47 patients) with subjective cognitive concerns.^12^ Importantly, self-reported cognitive problems are not only common but are also related to well-being for people with FND. In one study in 61 patients with clinically established functional movement disorder, the authors noted that cognitive concerns were associated with reduced health-related QoL (*r* = −0.72),^13^ while a second paper in 62 patients with FDS similarly found a significant correlation between perceived cognitive difficulties (*r* = −0.40) and QoL.^14^

Adding to subjective concerns, there has been some work using performance-based cognitive testing paradigms in FND. One systematic review and meta-analysis of 84 total studies (8,654 participants) investigated objective cognition in FDS compared to healthy controls and epilepsy. Results showed worse test scores in patients with FDS versus healthy controls (35 studies), with at least moderate effect sizes in all primary cognitive domains (attention/processing speed, language, visuospatial skills, learning/memory, executive functioning, social cognition, global cognition; overall Hedge’s *g* = −0.61^15^), which correlated with reduced QoL.^16^ With respect to the epilepsy comparison (57 studies), findings indicated a small advantage in the FDS group (Hedge’s *g* = 0.17), driven by a focal deficit in language in patients with left hemisphere temporal lobe epilepsy. Meta regressions examining predictors including demographics, psychiatric diagnoses, and antiseizure medications did not show significant associations with cognition, although variables such as type/severity of mental health symptoms and other psychoactive medications were not investigated^15^ (and may influence cognition in FND).

In contrast to FDS, the literature on cognitive test performance in other FND subtypes is sparse. In one systematic review of 56 total studies (2,260 participants), the authors reported mixed evidence, with some studies suggesting worse cognitive test performance in an FND group and others finding no difference.^17^ However, only a few studies specifically reported on this question in functional movement disorder or functional cognitive disorder (FCD). This may suggest that performance-based cognitive deficits are more pronounced in FDS versus alternative functional presentations, although it could also simply reflect the relative lack of empirical literature in other subtypes.

Beyond reporting on results from questionnaires versus cognitive testing separately, there is some interest in evaluating discrepancies between the 2 measurement modalities in FND.^18^ In a review of 18 studies on relationships between subjective and objective measures of distress and arousal in FND, only 4/18 studies found significant correlations, leading the authors to suggest that the discordance itself may have clinical utility (eg, for diagnosis and/or treatment).^19^ However, in the domain of cognition, a non-relationship between self-report and performance-based outcomes is a transdiagnostic feature of neuropsychiatric disorders rather than being specific to FND. That is, a recent umbrella review synthesizing data from 50 individual review papers across >20 different clinical populations (including FND) found that subjective and objective cognition are largely non-overlapping constructs (<2% variance shared), regardless of the particular disorder being investigated or assessment tool being used.^20^ This suggests that the lack of an association between a patient’s report of their cognitive problems and their scores on specific cognitive tests is not unique to FND, and the underlying mechanisms of cognitive dysfunction in FND are likely more complex.

Taken together, the literature shows frequent cognitive problems in FND that are not exclusive to patients diagnosed with FCD.^21^ This suggests the possibility of a functional cognitive overlay, characterized by core impairments in attention and processing speed that mitigate higher-level aspects of cognition (eg, memory, executive functioning) and prevent the patient from engaging fully in important social and occupational activities.^15^^,^^18^ Nevertheless, cognitive difficulties in FND are often not well recognized outside the context of FCD. For example, broad, interdisciplinary recommendations for outcome assessment in FND have neglected cognition as an important construct to measure.^22^ Moreover, the majority of available treatments target sensorimotor issues (eg, seizures, tremors) and/or co-occurring mental health conditions^23^^–^^25^ rather than cognition. Although psychotherapy is sometimes associated with statistically significant improvements in cognition for patients with FND, the effect sizes are small and may not be clinically meaningful.^12^^,^^26^ Overall, healthcare providers are increasingly recognizing that frequent cognitive difficulties limit the potential for treatment gains and reduce QoL for patients with FND, reflecting a gap in the scientific literature.^20^^,^^27^^,^^28^

**Figure 1.**
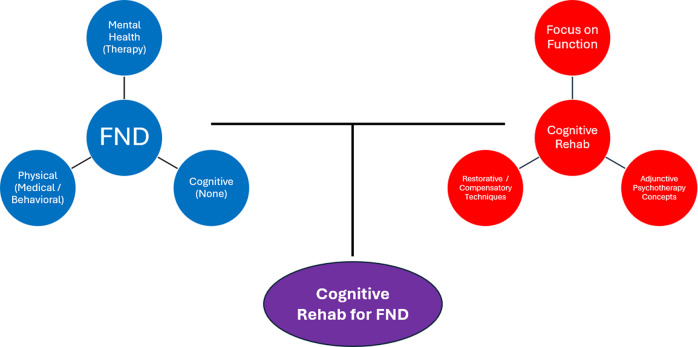
A conceptual diagram representing FND and cognitive rehabilitation as having relatively separate scientific literatures and independent domains of clinical practice. The upper left shows 3 key symptom dimensions in FND: mental health (eg, depression, anxiety, traumatic stress, dissociation), which is often treated with psychotherapy; physical (eg, fatigue, pain, sleep), which can be treated with medical (eg, pharmacological) or behavioral (eg, sleep hygiene) techniques. Cognitive symptoms have historically been untreated in FND. The upper right shows 3 major influences on cognitive rehabilitation: a strong focus on everyday functioning as the primary outcome, use of restorative and/or compensatory techniques, and infusion of principles from psychotherapy (eg, therapeutic alliance, emotion-cognition link, coping strategies, insight/awareness raising). The bottom shows the purpose of the current paper: bringing together an unmet need in FND (untreated disabling cognitive problems) and a widely available transdiagnostic intervention framework (cognitive rehabilitation).

In this review, we outline the need for evidence-based cognitive interventions that can be applied across the diverse family of FND phenotypes, as well as the rationale for why a particular set of approaches known as *cognitive rehabilitation* is well-suited to this population (see Box 1; Figure 1). We describe the nature of cognitive problems in FND, as well as specific rehabilitation techniques that have the potential to successfully address these issues. We highlight unique challenges and pitfalls to be addressed. We emphasize clinically actionable advice and guidance regarding best practices for adapting and applying cognitive rehabilitation for FND patients (Box 2; Table 1), as well as future directions for scientific investigation in this area.

Box 1: Rationale for cognitive rehabilitation in FND.

1. Cognition is underrecognized and undertreated in FND.^16^^,^^28^ Currently, there are no evidence-based interventions specifically tailored to treat cognitive problems across FND subtypes, in spite of the frequency and severity of these symptoms. Cognitive rehabilitation is the most well supported type of intervention to address this issue.^29^
2. Cognitive rehabilitation is a transdiagnostic approach that transcends a specific brain disease, disorder, or symptom profile. It has empirical support for improving cognition and everyday functioning across a wide range of clinical disorders in neurology and psychiatry.^30^^–^^33^ FND is the quintessential mind-brain-body neuropsychiatric disorder,^1^ making it well suited for a broad-based treatment.
3. Primary outcomes targeted in cognitive rehabilitation are everyday functioning and well-being. FND is associated with high rates of chronic disability^5^ and has equivalent or greater impacts on quality of life than do other neurological disorders.^70^ Core functional symptoms (eg, seizures, gait disturbance) are often persistent and do not always respond to treatment, but patients with FND can still experience better quality of life, even while living with functional symptoms. A treatment with the explicit goal of enhancing functional independence and helping patients engage meaningfully in their lives is likely to be successful in FND.
4. Psychotherapy is one of the best treatments for FND and its mental health comorbidities,^23^^–^^25^ but cognitive problems often limit engagement in psychotherapy for functional symptoms, serving as a barrier or perpetuating factor that prevents healing and recovery. For some patients, an initial focus on ameliorating inattention, slowed processing speed, forgetfulness, and/or other cognitive difficulties can facilitate fruitful engagement in psychotherapy.

Box 2: General advice and tips for using cognitive rehabilitation in FND.

- **When possible, conduct a neuropsychological assessment, using the biopsychosocial framework,** ^66^^,^^71^ **prior to treatment.** The evaluation can serve multiple purposes, such as (i) helping the patient build insight into their own cognitive functioning; (ii) identifying relevant predisposing, precipitating, perpetuating, and protective factors that guide intervention; and (iii) facilitating a better understanding of the patient’s cognitive profile (ie, strengths and weaknesses), to inform selection of optimal treatment strategies.
- **Begin all cognitive rehabilitation interventions with clear, direct, and compassionate psychoeducation about FND** that (i) meets the patient where they are at, (ii) clearly defines and describes FND, (iii) emphasizes the mind-brain-body connection, (iv) highlights the pervasiveness of cognitive problems in FND, and (v) validates the authenticity of the patient’s symptoms. Be responsive to the patient’s body language and comments during initial psychoeducation. Prepare to discuss common patient questions such as, “is it all in my head?” and “what should I do when people tell me I’m faking?”
- **Prioritize the therapeutic alliance throughout treatment.** Acknowledge any prior FND-associated iatrogenesis experienced by the patient.^72^ Make room in treatment for processing emotions and highlight the link between emotions and cognitive functioning. **Utilize relevant techniques from psychotherapy** such as behavioral activation (cognitive behavioral therapy) and/or cognitive defusion (acceptance and commitment therapy).
- Co-create *SMART treatment goals with the patient (and their family, if appropriate), based on their value system, together with results of their neuropsychological evaluation (eg, cognitive strengths and weaknesses).
- **Assess the patient’s awareness of their cognitive strengths and weaknesses and their current use of external aids** (eg, writing to-do lists, setting alarms, using a calendar) **early in treatment,** to inform selection of rehabilitation strategies and tactics.
- **Measure relevant self-reported and/or performance-based cognitive outcomes at multiple timepoints during the treatment protocol. Share results openly with the patient and family and discuss any changes that are observed over time.** This is consistent with a transparent approach to assessment and diagnosis in FND generally^6^ and FCD, specifically.^36^ Tracking key outcomes and discussing the results is considered a component of patient-centered care in FND and can help with the refinement of a cognitive intervention by identifying techniques that are particularly effective for the patient.
- **Consider using rehabilitation techniques that target inattention,** ^73^^–^^75^ **slowed processing speed,** ^76^ **and metacognitive errors,** ^77^ given the high frequency of these issues in patients with FND.^15^^,^^18^
- **Identify and target specific functional cognitive symptoms that are common and associated with distress and impairment.**
  - **Avoidance of mental effort (cogniphobia):** Avoidance can prevent contact with positive reinforcers and perpetuate anxiety and disability. Behavioral treatments for this issue focus on exposure to the feared stimulus, and cogniphobia is no different. Exposure will typically involve small action-oriented experiments that allow for the experience of accomplishment and begin to challenge maladaptive schemas related to brain damage and cognitive disability.
  - **Memory perfectionism and hypervigilance to cognitive lapses:** Intolerance of minor cognitive lapses and monitoring for/hyperfocusing on cognitive mistakes can lead to significant distress and impairment. Therapeutic techniques may include psychoeducation on the frequency of cognitive lapses in the general population,^78^ identifying maladaptive thinking styles (eg, via thought records),^49^ and/or mindfulness exercises.^74^
  - **Overreliance on compensatory strategies:** Compensatory strategies can be a double-edged sword in FND and a discussion about pros and cons (with the patient’s active participation) can be constructive. For example, writing a to-do list can have advantages of offloading information from prospective memory and remaining goal oriented. However, multiple sticky notes with upcoming appointments, goals, and other tasks stored all over the home can be overwhelming and counterproductive. Treatment may focus on finding a “Goldilocks” zone, in which the patient uses these strategies only when they are adaptive and productive.
- **Address other common and disabling symptoms in FND such as fatigue and chronic pain.** ^8^^,^^11^ Directly address these issues when necessary (prior to or concurrent with cognitive rehabilitation), ideally via integrated care teams utilizing evidence-based treatments.^79^

*SMART = Specific, Measurable, Achievable, Relevant, Time-Bound.

**Table 1.** Cognitive Rehabilitation Approaches Recommended for Consideration in Patients with FND

Technique (example citation)	Brief description	Rationale for use in FND
Attention process training (Lopez-Luengo and Vazquez)^a^ ^73^	Trains 4 aspects of attention (sustained, selective, divided, and alternating) via practicing of tasks, such as cancellation, multilevel card sorting, and serial subtractions.	Attentional dysfunction appears to be a foundational symptom dimension in FND^15^^,^^18^^,^^80^^,^^81^ and patients may avoid cognitive effort (eg, concentration).^18^^,^^36^^,^^39^ An intervention that promotes intentional, mindful engagement in attention training may help (i) remediate a central underlying deficit that holds back other cognitive processes and (ii) counteract behavioral avoidance, providing evidence of small successes in simple cognitive activities (similar to exposure for anxiety disorders).
Mindfulness (Smart et al.)^74^	Involves deliberate direction of one’s attention to the present moment without judgment.	Mindfulness can be associated with improvements in moment-by-moment attention, potentially counteracting distractibility in FND. Moreover, the cognitive milieu of openness, acceptance, and equanimity can benefit these patients, who often struggle with poor self-concept, low self-awareness, stigma, negative expectations, and various trauma and stressor-related disorders.^8^^,^^9^
LEAP for conversational attention (Huckans et al.)^75^	An acronym (Listen actively, Eliminate distractions, Ask questions, Paraphrase) serving as the basis for improving focused attention in conversations with other people.	Patients with FND may have trouble connecting with other people, potentially due in part to inattention and distractibility. Simple mnemonics such as LEAP are easy to learn, can be practiced in treatment, and can be readily applied to everyday life, in order to improve social interactions and relationships.
Time pressure management (Winkens et al.)^76^	Aims to facilitate awareness and acceptance of slowed processing speed and teaches patients to plan ahead and allow adequate time to complete tasks.	Inefficiencies in processing speed (including bradyphrenia) can be present in FND,^15^ and this can also be associated with slower execution of downstream cognitive tasks (eg, memory, problem solving). Related psychological symptoms such as reactivity, frustration, and impersistence (“giving up”) are common. Treatment oriented toward nonjudgment in the face of one’s limitations, coupled with compensatory training (ie, future planning) to support task completion, is likely to be beneficial.
Procedural/habit learning (Oudman et al. 2015)^82^	Capitalizes on implicit memory systems to compensate for reductions in declarative memory. For example, “linking” strategies wherein a novel behavior (eg, a phone call to a friend) is linked to an overlearned behavior (eg, brushing teeth) via an external cue (eg, a sticky note reminder next to the sink).	Many patients with FND perceive major memory problems in their everyday lives.^11^ A substantial portion of these patients show difficulties on tests of episodic memory.^15^ Implicit (procedural) memory systems are independent of explicit systems and are often robust in the face of dysfunctional neural networks, even in people with severe amnestic syndromes (eg, Alzheimer’s disease, Korsakoff’s syndrome). This can allow patients with FND to take advantage of preserved habit learning to gain back lost functioning in their lives.
Metacognitive strategy training (Sella et al.)^77^	A broad category of approaches to improve “thinking about thinking”—the ability to accurately understand and appreciate one’s own cognitive functioning.	Poor metacognition has been advanced as a potential mechanism underlying functional cognitive symptoms.^42^ It is believed to cause or exacerbate common issues such as poor memory self-efficacy, underestimating cognitive abilities, and self-fulfilling prophecies of cognitive failure and dysfunction. Targeting metacognition may be one of the most direct and comprehensive approaches to cognitive rehabilitation in FND.^42^

*Note:* When possible, the recommendations provided in this table are based in empirical literature. In the absence of a well-developed evidence base, we extrapolate from work in related disorders (eg, chronic pain, persistent symptoms after concussion) and we utilize our own clinical experience, as well as that of our colleagues (eg, published clinical recommendations), to formulate the best advice we believe to be currently available.

a Attention process training tasks may require modification (ie, reduced complexity/difficulty) to fit the capacity and emotional state of the patient.

## Foundations of cognitive rehabilitation

Cognitive rehabilitation reflects a diverse set of strategies and tactics that share the goal of helping patients with cognitive difficulties engage meaningfully in their lives (eg, work, school, hobbies, relationships).^29^ Most conceptual models divide cognitive rehabilitation into restorative versus compensatory approaches. Regarding the former, a specific cognitive deficit is directly targeted and trained, in order to enhance that particular function (eg, via computerized working memory training), thereby providing the patient with more “mental horsepower” with which to accomplish everyday activities. On the other hand, compensatory techniques acknowledge that the impairment itself may not be amenable to remediation, and instead teach alternative tactics that can allow the patient to achieve the same objective via a different pathway. Compensatory cognitive rehabilitation is analogous to using crutches for improvement of mobility after a lower limb injury and often involves utilization of practical tools and external aids, such as a digital memory notebook, alarms, cues, and/or calendars in daily life.

Cognitive rehabilitation is well known for its use in patients with TBI and stroke,^29^ but it is fundamentally transdiagnostic, with evidence showing improvements in cognitive and functional outcomes for a diverse set of clinical populations, including mild cognitive impairment,^30^ long COVID,^31^ and PTSD,^32^ among others. Specific exercises may focus on a particular cognitive domain (eg, attention, language, memory, executive functions) and/or a functional task (eg, making dinner, checking email, paying bills, organizing medications, interacting with healthcare providers). Treatment can be either individual or group based and relies on cross-cutting principles such as provision of psychoeducation, co-creation of shared goals with patients and families, personalization of the intervention to the patient’s situation and level of cognitive functioning, internalization of skills (automaticity), and generalization of learned material to new contexts and situations. Additionally, although cognitive rehabilitation is distinct from traditional psychotherapy, experienced practitioners explicitly acknowledge and discuss the close link between cognition and emotion, with a focus on building the therapeutic alliance using tools such as active and empathetic listening, motivational interviewing, and creating space for processing of emotions during a session.^29^ This emphasis has even led to the development of several combined psychotherapy/cognitive rehabilitation interventions (eg, for schizophrenia^33^ and PTSD^32^), which have shown promise. Overall, we view cognitive rehabilitation through a broad, nonreductive lens that extends beyond exclusive reliance on drill and practice approaches (eg, computerized training to enhance working memory) or calendar use (eg, to compensate for reduced episodic memory). In our view, cognitive rehabilitation is a flexible person-centered and biopsychosocial framework that capitalizes on an entire armamentarium of techniques to target cognitive functioning, with the ultimate goal of improving quality of life and participation in meaningful activities of daily living for patients with neuropsychiatric disorders, including FND.

## The nature of cognitive problems in FND

Although the frequency and severity of cognitive difficulties in FND is clear, underlying mechanisms and perpetuating factors are still being investigated. For example, patients with FND tend to have disrupted interoceptive awareness (eg, reduced accuracy in monitoring their own heart rate^34^ and breathing)^35^, which has been proposed in a conceptual model^18^ to be related to observed deficits in focus and concentration.^15^ One potential pathway for this effect may be excessive attention toward and worry about the patient’s own body and/or brain, which could limit external, goal-oriented behavior and interfere with full engagement in cognitive tasks, although this hypothesis has not yet been directly tested. Additional factors with relevance to FND include memory perfectionism, catastrophizing in response to minor errors, and hypervigilance to cognitive lapses.^36^ Elevated memory perfectionism has been shown to differentiate patients with functional memory disorder from healthy comparisons^37^ and is associated with severe memory complaints (after controlling for objective cognitive performance and depression) in patients with persistent cognitive symptoms following mild TBI.^38^ Catastrophizing as a relevant mechanism in FND has been extrapolated from related conditions such as chronic pain and prolonged symptoms after mild TBI,^39^^,^^40^ while, to our knowledge, hypervigilance to cognitive errors has not yet been directly tested.^36^ The nocebo effect (expectations for future illness setting the stage for dysfunction and disability) is believed to be core to the onset and maintenance of FND in general^41^ and can be associated with failures of metacognition^42^ that are central to patients’ lived experiences of cognitive impairments. Relatedly, some people with FND go to extreme lengths to avoid cognitive effort (overestimating the severity of their deficits), leading them to overrely on compensatory strategies for simple tasks, which may perpetuate their symptoms over time.^36^

As noted above, mental health comorbidities are common in FND, with co-occurring mood, anxiety, somatization, and PTSD symptoms serving as additional possible contributors to cognitive issues.^16^ Of particular relevance, dissociation—characterized by loss of control or awareness of cognitive or physical processes—is elevated in FND.^43^ Although the long-term relationship between dissociation and cognitive functioning in FND is not yet clear, it is likely that acute fluctuations in alertness and attention resulting from experiences such as depersonalization and derealization are related to patients’ perceptions of memory loss and confusion. Indeed, dissociation is considered by some researchers to be core to the phenomenology of FDS,^44^ and one study found that the paroxysmal events in FDS can temporarily interfere with engagement in formal cognitive testing, although this appears to occur only rarely.^45^

**Figure 2.**
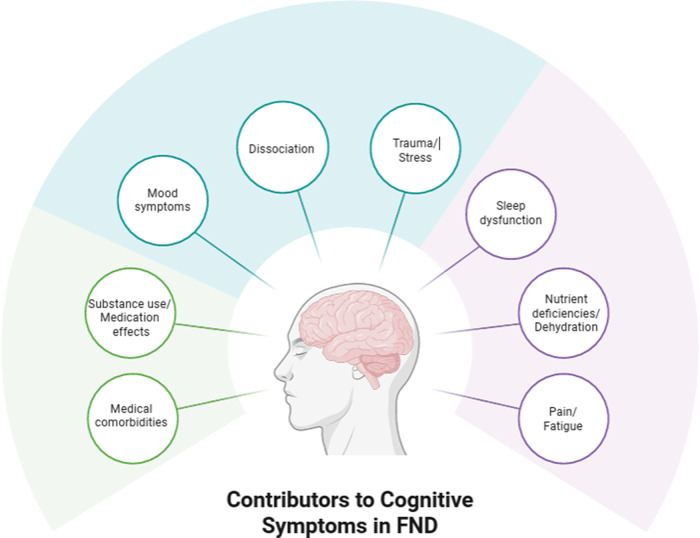
Hypothetical visual representation of factors to consider in the application of cognitive rehabilitation in patients with FND. The model is not intended to be exhaustive but rather to provide examples of several of the most common influences on subjective and objective cognitive functioning in FND. The mechanisms through which each factor relates to cognition (ie, the solid lines connecting the contributors to the individual with FND) are complex and may vary from person to person. A key proposed feature is disrupted attention, which can serve as a bottleneck that mitigates other cognitive abilities (eg, memory, executive functioning) and may result from any or all of the factors listed in the figure.

Adding to psychological conditions influencing cognition in FND, physical states such as fatigue, pain, poor sleep, and medication effects, are common^8^^,^^46^^,^^47^ and are believed to be associated with reduced attention, possibly driving downstream difficulties in other cognitive domains as well.^48^ In order to account for complex relationships among various biopsychosocial influences on cognition in FND, several conceptual frameworks have been proposed.^36^^,^^48^^,^^49^ However, at present, these proposals are grounded primarily in basic statistical approaches (eg, bivariate correlations or multiple regressions) and lack quantitative support from detailed structural equation models or network analyses of cognition in FND. In light of the developing literature in this area, we provide a simple and clinically oriented hypothetical visual representation of relevant factors to consider when assessing and treating cognition in FND (Figure 2).

## Applying cognitive rehabilitation to FND

The promise of cognitive rehabilitation in FND is supported by its efficacy in related conditions that share similar risk factors, underlying mechanisms, and clinical symptoms, such as prolonged cognitive difficulties following mild TBI.^50^^,^^51^ For example, in one study of 126 U.S. military service members with chronic symptoms following mild TBI, participants were randomly assigned to one of 4 interventions: (i) psychoeducation, (ii) self-administered computerized cognitive training, (iii) therapist-directed manualized cognitive rehabilitation, or (iv) therapist-directed cognitive rehabilitation integrated with cognitive behavioral therapy.^52^ Therapist-directed cognitive rehabilitation demonstrated superior self-reported daily cognitive functioning (via the Key Behaviors Change Inventory)^53^, with 23% of participants showing reliable gains on this measure compared with computer-based training (0%) and psychoeducation (7%), while integration of cognitive behavioral therapy offered no additional benefit (18%).^52^ Additional data were provided by a meta-analysis demonstrating a small positive effect of cognitive rehabilitation on neuropsychological test performance among service members with chronic symptoms following mild/moderate TBI (*d* = 0.22),^54^ although findings are limited by the lack of reporting on subjective cognition. Common strategies highlighted across these studies and by field experts include careful use of language to minimize iatrogenic effects, psychoeducation, promotion of self-efficacy, focus on daily function, and individualized treatment targets.^55^

The literature specifically investigating post-treatment cognitive outcomes in FND is in its infancy. In one nonrandomized trial, changes in cognition were measured following neurobehavioral therapy, a 12-session multimodal psychotherapy for patients with FDS or epilepsy that incorporates a variety of elements such as motivational interviewing, psychoeducation, cognitive behavioral therapy, self-awareness/mindfulness, and self-management, among others.^25^ In 47 patients with FDS and TBI, neurobehavioral therapy was associated with statistically significant reductions in subjective cognitive concerns, based on a ~7-point improvement on the Quality of Life in Epilepsy Inventory–31 (range = 0–100).^12^ In a smaller subset of 37 patients with FDS and TBI from the same study, the treatment was related to modest pre-post increases in performance on the Montreal Cognitive Assessment screening test (approximately 1.5 points out of a total score of 30).^26^ Similarly, a feasibility trial investigating a 5-session online acceptance and commitment therapy group for FCD (44 participants randomized) showed a trend toward a modest post-treatment improvement in mental flexibility as measured by the Acceptance and Action Questionnaire II (ie, a change of 2.8 points at 6-month follow-up on a 7–49-point scale).^56^ Lastly, in a pilot randomized controlled trial in patients with FCD following head injury, 11 sessions of cognitive rehabilitation (*n* = 13) or cognitive-behavioral therapy (*n* = 11) produced comparable improvements in the primary outcome measure – the Multifactorial Memory Questionnaire (MMQ), Satisfaction scale.^40^

Taken together, preliminary findings (based primarily on pilot or feasibility studies) raise the possibility that various interventions may confer some benefit for cognitive problems in FND. However, effect sizes to date have been modest and only one of the 3 studies utilized cognitive rehabilitation (*n* = 13), which was considered the control group rather than the experimental intervention.^40^ The other 2 interventions were psychotherapies (neurobehavioral therapy and acceptance and commitment therapy), which have relevance to cognitive rehabilitation but are not specifically designed to target domains of cognition and everyday functioning in the same manner. That is, although some tools and processes from psychotherapy are undoubtedly useful adjuncts in cognitive interventions, the cognitive benefits from these approaches are typically indirect (eg, via supporting the therapeutic alliance and reducing comorbid mental health symptoms), likely leading to smaller effect sizes. Importantly, we are not aware of any cognitive rehabilitation interventions developed or adapted for FND and systematically evaluated in a fully-powered clinical trial. Based on our review of the literature on diverse cognitive rehabilitation techniques that are available, together with the unmet needs for addressing cognitive difficulties in FND, we believe that there is unrealized potential of this intervention approach for many patients.

In the absence of an evidence-based cognitive rehabilitation protocol that is tailored to FND, we contend that many patients can benefit from the thoughtful application of transdiagnostic rehabilitation techniques, grounded in parallel literature in related neuropsychiatric conditions, to address their cognitive and functional limitations. This is supported by evidence suggesting that many patients with FND communicate their cognitive problems in everyday life via self-report questionnaires^11^^–^^14^ and that at least some of them demonstrate cognitive deficits on objective neuropsychological tests.^15^^,^^17^ Moreover, based on our collective clinical experiences and conversations with colleagues, we have also heard many firsthand accounts of disabling cognitive problems from patients with FND and their loved ones. Thus, we expect that qualitative research investigating the lived experiences of cognitive difficulties in FND would be instructive in applying cognitive rehabilitation techniques. Although there is a burgeoning literature using qualitative methods to uncover details of stigma, iatrogenesis, and overall symptom burden in FND,^9^^,^^57^^–^^61^ none of these studies to date have focused specifically on cognition, leaving a gap in the literature.

With this in mind, we delineate fundamental concepts and principles, based on our review of the scientific evidence in related disorders and our clinical experience. Box 2 provides actionable advice and tips that are frequently useful during cognitive rehabilitation in patients with FND. Additionally, Table 1 provides a nonexhaustive list of specific tools and protocols within the cognitive rehabilitation framework, which we believe have potential for use in some patients with FND.

## Challenges and pitfalls

Access to care remains a major challenge for patients with FND. Neuropsychologists are uniquely positioned to deliver cognitive rehabilitation, but availability of these services is limited and advancing treatment access will require contributions from multiple disciplines. Whereas there are approximately 5,000 neuropsychologists in the United States,^62^ there are an estimated 170,000 speech-language therapists and 140,000 occupational therapists.^63^ Ratios are likely similar in other Western nations. Moreover, clinical psychologists, social workers, and other interventionists can be trained to deliver cognitive rehabilitation, meaning that dissemination and utilization of these approaches is inherently multidisciplinary. However, as depicted in Figure 1, the FND and cognitive rehabilitation communities have historically been isolated from each other, which we believe has contributed to the lack of access to treatment for patients with FND.

Critically, the training needed to competently deliver cognitive rehabilitation is not standardized or regulated and will likely differ based on the provider’s specialty. That is, clinicians with a background in FND and mental health/psychotherapy (eg, clinical psychologists, social workers) may need education in cognitive rehabilitation, such as the textbook^29^ and courses available through the American Congress of Rehabilitation Medicine (https://acrm.org/meetings/cognitive-rehab-training/). Meanwhile, providers such as speech-language and occupational therapists who are familiar with FND will likely benefit from an introduction to behavioral interventions, possibly through books and workshops on cognitive behavioral therapy^64^ and/or acceptance and commitment therapy^65^ (see https://www.pesi.com for courses). With more diverse interventionists receiving the specialty training needed to offer cognitive rehabilitation, there will be increasing opportunities for patients with FND to access this needed service, supplementing interventions which are already available.^66^^–^^68^

Even if a treatment provider is available, one potential argument against using cognitive rehabilitation in patients with FND is that the FCD subtype and its corresponding functional cognitive overlay can be associated with a pathological overuse of compensatory strategies.^36^ In other words, some of these patients have low cognitive self-efficacy, poor metacognitive awareness, and hypervigilance toward cognitive errors that can lead to an avoidance of mental effort (eg, cogniphobia)^69^ and an overreliance on methods to compensate for presumed cognitive impairments. For example, patients may create a grocery list for 2 items, write sticky note reminders for routine tasks such as brushing their teeth, or set alarms to remind themselves to use the bathroom (in the absence of urinary incontinence). In such cases, an interventionist who encourages increased implementation of external aids without consideration for the patient’s specific metacognitive problems may be exacerbating the issue. Fortunately, we believe that this is an excessively narrow and rigid conceptualization of cognitive rehabilitation, meaning that effective treatment does not require the unfettered promotion of external aids in all patients or all situations. Instead, we recommend that clinicians aim for *refinement of compensatory strategy use to adaptive and healthy levels.* This may include increased utilization in some patients or in some contexts, with decreased use in others. Moreover, as noted in Table 1 and the Case Vignette, some cognitive rehabilitation strategies (eg, attention process training, mindfulness, behavioral experiments) can be leveraged to directly address the problem of avoiding cognitive effort through overuse of compensatory techniques.

## Case Vignette

Ms. Smith is a 52-year-old White woman with a college degree, employed as a receptionist, who undergoes a neuropsychological evaluation for memory concerns. During the interview, she expresses certainty that she is developing Alzheimer’s disease (“I’m sure of it”), a condition that she observed impact her grandmother (diagnosis made at age 78). The evaluation reveals significant avoidance of tasks requiring episodic or prospective memory due to the belief that her “brain is broken,” as well as a persistent fear of failure. She reports frequently reading news articles and books about signs of early Alzheimer’s disease, worsening her fear and anxiety. Performance-based test results are variable and inconsistent with a typical amnestic syndrome (ie, she performs worse on recognition compared to retrieval memory). She is diagnosed with FCD, provided with psychoeducation, and referred for cognitive rehabilitation.

For this patient, the interventionist works collaboratively to create shared goals around improving FCD symptoms, including memory and everyday functioning. The clinician follows a basic structure that emphasizes ongoing psychoeducation, self-efficacy, monitoring memory successes, refining the use of compensatory strategies, and implementing behavioral experiments. Goals of treatment include (i) understanding different types of memory lapses and their causes, (ii) cultivating the healthy use of strategies to improve memory performance in daily life; (iii) reducing perceived threats associated with engaging in everyday activities requiring memory; and (iv) increasing overall cognitive self-efficacy. Below, we present a non-exhaustive set of cognitive rehabilitation modules that may be applied in FND, including examples of specific therapeutic activities for Ms. Smith, mapped onto the content from Table 1 (where relevant).

### Psychoeducation and motivation to engage in treatment

Even if a patient has received initial psychoeducation prior to the referral for cognitive rehabilitation, we believe that there is benefit to beginning with a check-in regarding their overall health literacy and specific understanding about cognition in FND. This approach provides a basis for knowledge sharing that facilitates co-creating a tailored treatment protocol. Generally, this includes reinforcing the biopsychosocial model of FND and discussing mechanisms contributing to cognitive problems. Different types of memory difficulties and their causes (eg, Alzheimer’s disease, FCD) can be provided, with emphasis on the validity and realness of FCD symptoms. The interventionist and patient then work collaboratively to develop an understanding of how symptoms are manifesting in everyday life and what may be perpetuating the cognitive problems.

*Module 1: Ms. Smith and the interventionist discuss how her fear of Alzheimer’s disease is leading to hypervigilance to cognitive errors, and how the notion, “my brain is broken,” is a catastrophic thinking pattern that contributes to her anxiety and distress. Evidence for and against Alzheimer’s disease is identified. Differences between an amnestic memory disorder such as Alzheimer’s disease and an attentional disorder such as FCD are reviewed in order to set the stage for effective treatments (e.g., Attention Process Training, mindfulness, Table 1). Avoiding reliance on her own memory (e.g., writing down a list of 2 items to buy at the grocery store) is highlighted as possibly perpetuating her cognitive difficulties. The interventionist describes cognitive rehabilitation in general terms and uses motivational interviewing techniques to elicit change talk in Ms. Smith as she considers her willingness to engage in treatment. Homework includes brainstorming reasons for (and barriers to) making the decision to move forward with treatment, which will be discussed in the next session.*

### Collaborative goal setting

The interventionist presents general features of metacognition (including metacognitive errors) with the patient (Table 1). This informs treatment planning, which proceeds via the establishment of Specific, Measurable, Achievable, Relevant, and Time-Bound (SMART) goals. Each aspect of the SMART framework is reviewed and applied to the patient’s situation and context, often in the form of a worksheet. An expectation is set for regular brief outcome assessment to facilitate quantifying progress toward treatment goals (the “M” in SMART).

*Module 2: The treatment plan is co-created. Ms. Smith identifies 2 overarching SMART goals of improving her memory and reducing her distress/worry about Alzheimer’s disease. She is encouraged to discuss options for measuring change and specific benchmarks that can be used to highlight progress. Questionnaires are selected to assess the constructs of interest throughout treatment (i.e., MMQ-Satisfaction, MMQ-Ability, Beck Depression Inventory, Beck Anxiety Inventory). Next, Ms. Smith engages in brainstorming about specific changes that would support her 2 primary goals. For example, she reports taking copious notes during most conversations because of concern that she will forget what was said. Her friends and family have told her that this feels awkward to them (as if they are “being interviewed”) and she seems distracted by her note taking rather than engaged in the conversation. A short-term objective is established to have a brief conversation with her closest friend without taking notes and then, during the subsequent treatment session, to report on how she felt during this exercise and what she could recall about the conversation (emphasizing the gist over details).*

### Compensatory strategies

Reviewing the pros and cons of different compensatory strategies can clarify those that are facilitators versus hinderances to healthy functioning. Careful examination of these strategies is conducted collaboratively. Unhelpful strategies (eg, dozens of sticky note reminders) can be identified, while alternatives (eg, using a single calendar judiciously) are encouraged.

*Module 3: Advantages and disadvantages of various compensatory strategies are discussed and outlined via a worksheet. The concept of a “Goldilocks” approach to using these strategies (the correct amount of the right strategies) is offered by the interventionist. The notion of habit learning is reviewed, including occasional use of “linking” strategies (Table 1). Ms. Smith works to capitalize on her intact implicit memory and provide herself with helpful reminders for unusual tasks (e.g., purchasing and mailing a birthday card), thereby supporting her prospective memory. She works on acknowledging and accepting that she has the capacity to perform simple tasks (e.g., writing a letter) once she remembers to do it. For homework, she tracks the use of helpful and unhelpful compensatory strategies, with an effort to reduce the latter.*

### Enhancing attention and processing speed

Many people with FND focus on memory loss, although inefficiencies in attention and processing speed appear to be a bottleneck and driver of downstream cognitive difficulties for many patients. Thus, following psychoeducation, rapport building, a commitment to engage in treatment, and acknowledgement of which compensatory strategies are and are not helpful, the interventionist can pivot toward approaches designed to ameliorate issues of slowed thinking and distractibility.

*Module 4: Ms. Smith is provided with education about various strategies to enhance focus and concentration. Based on her individualized treatment plan, the interventionist introduces the utility of attention toward the present moment and leads a brief mindfulness exercise during the session (Table 1). Ms. Smith talks about her experience of and reactions to the exercise. They also discuss the LEAP technique for conversational attention (Listen actively, Eliminate Distractions, Ask questions, Paraphrase; Table 1), which dovetails with work from Module 2 about reducing note taking during conversations with friends and family. For homework, Ms. Smith agrees to complete several short mindfulness exercises independently and to try out the LEAP approach (without note taking) during upcoming social interactions.*

### Behavioral experiments

Engaging in simple, realistic behavioral experiments can challenge assumptions about cognition, identify barriers to cognitive functioning, and promote experienced successes (ie, exposure therapy). This approach can be considered under the umbrella of insight raising and metacognitive training in that a small change in behavior leading to the experience of achieving a goal (eg, remembering a short grocery list) can alter maladaptive thinking patterns and ingrained schemas (Table 1).

*Module 5: Ms. Smith reports using detailed written instructions to make her pot of coffee every morning. While this eases her worries about making errors, it is likely reinforcing the belief that her “brain is broken.” She sets a goal of attempting to prepare her coffee one time without looking at the written instructions (i.e., homework for the upcoming week). She uses mindfulness and deep breathing techniques to cope with anticipatory anxiety before this exercise. She finds that she is able to make the coffee without any trouble and is surprised with the ease in which she completed the task unsupported.*

### Wrap-up and future planning

At the conclusion of any cognitive rehabilitation protocol, it is helpful to summarize accomplishments to date, review progress toward the overarching SMART goals, and plan for the future. A discussion about the need for ongoing work outside of formal treatment is typically beneficial. Future homework assignments (potentially framed as daily habits or routines) can be discussed and a booster session in 1–2 months may be offered.

*Module 6: Ms. Smith is encouraged to talk through her progress during treatment. Assessment data are discussed and successes, barriers, and challenges are reviewed. Ms. Smith engages in future planning, including updating her SMART goals for application in her daily life and setting reasonable expectations for regular engagement in mindfulness, metacognitive worksheets, and behavioral experiments. A booster session is scheduled for 2 months in the future.*

## Conclusion

The broader community of FND researchers and clinical care providers have only recently begun to appreciate the frequency and extent of cognitive problems in this condition. Theoretical mechanisms have been proposed and are still a matter of ongoing debate and investigation. Advances in FND interventions to date have largely focused on other (non-cognitive) symptom dimensions, leaving a gap in the armamentaria of providers who wish to address disabling cognitive problems in their patients. Therefore, we recommend that future researchers work to develop and disseminate an evidence-based cognitive rehabilitation protocol specifically designed for patients with FND and their families. The protocol should be informed by the scientific literature, including results from (i) performance-based neuropsychological testing, (ii) self-report questionnaires, and (iii) qualitative interview data assessing the lived experiences of cognitive problems in patients with FND. In the meantime, we suggest that providers rely on transdiagnostic cognitive rehabilitation techniques, infusing relevant concepts from psychotherapy, in order to improve cognition and everyday functioning in their patients.
